# Early vs. Late Unplanned Returns to the Operating Room (URTOR) in Neurosurgery: Effect of Surgeon Experience and Complication Types

**DOI:** 10.3390/medicina61122117

**Published:** 2025-11-28

**Authors:** Mahmut Çamlar, Umut Tan Sevgi, Mustafa Eren Yuncu, Caglar Turk, Merve Oren, Berra Bilgin, Cafer Ak, Füsun Demirçivi Özer

**Affiliations:** 1 Department of Neurosurgery, Izmir City Hospital, University of Health Sciences, Izmir 35540, Turkey; umuttan1995@gmail.com (U.T.S.); fusun.ozer@sbu.edu.tr (F.D.Ö.); 2 Department of Public Health, Istanbul Faculty of Medicine, Istanbul University, Istanbul 34390, Turkey; meryemerve@gmail.com

**Keywords:** unplanned, reoperation, return to the operating room, neurosurgery

## Abstract

*Background and Objectives*: Unplanned return to the operating room (URTOR) is a sensitive indicator of surgical quality; however, data in neurosurgery are limited. This retrospective study analyzed patients who underwent URTOR following neurosurgical procedures over an eight-year period to define early and late patterns, identify underlying causes, and evaluate the distribution of cases according to surgeon experience. *Materials and Methods*: Records of 18,258 consecutive surgeries including both elective and emergency procedures in adult and pediatric patients, performed at a single center between 2010 and 2018 were retrospectively reviewed. Unplanned reoperations within 30 days of the index surgery were defined as URTOR; those occurring within ≤7 days were classified as “early,” and those occurring between 8 and 30 days were classified as “late.” Demographic data, surgical characteristics, causes of URTOR, and surgeons’ seniority were examined. *Results*: Among 18,258 neurosurgical procedures, 324 URTORs (1.8%) were identified. The median patient age was 38 years; 37% were children. Early URTOR comprised 59% and was primarily associated with hemorrhagic–vascular complications, whereas late URTOR accounted for 41% and was dominated by cerebrospinal fluid-related and infectious complications. Late events prevailed in significantly younger cases and were disproportionately followed by ventriculo–peritoneal shunt or endoscopic third ventriculostomy index operations. Junior surgeons performed 74% of later operations requiring URTOR versus 30% of early failures. Sex, weekday/weekend timing, and surgeons’ experience did not affect the overall URTOR classification categories. The median interval was six days. *Conclusions*: Centers worldwide have begun to examine URTOR rates, which are directly associated with hospital quality measurements. These results may inform targeted education and prevention by identifying patient groups at higher reoperation risk within a specific timeframe.

## 1. Introduction

Unplanned return to the operating room (URTOR) refers to unscheduled re-operation in the same center owing to any complication related to the index operation. The quality of surgical care is directly related to the URTOR rate, and the entire surgical team should aim to reduce this rate [1,2,3]. Additionally, the patient group with URTOR incurs an additional cost burden, causing distress to patients without health insurance. This undesirable condition also substantially prolongs hospital stay and negatively affects the quality of life of patients.

URTOR is more practical and determinative than accepted quality determinant markers, such as mortality rates. Mortality also occurs much less frequently than URTOR, and whether the results of mortality rates are reliable remains unclear [2,4,5]. Reported rates of URTOR in the neurosurgical literature vary considerably, ranging from 2.6% to 28% across different series [6,7,8,9]. URTOR in neurosurgery patients is an undesirable condition for the patient, companion of the patient, and the hospital management staff. As URTOR is an unexpected secondary intervention, patients and their families find it difficult to accept it.

URTOR also incurs additional costs for hospitals and insurance companies [10,11,12]. Therefore, each center should identify where the problem originated. Although addressing URTOR to reduce complications and increase patient quality of life is essential, few studies have reported URTOR rates, especially in neurosurgery care units. This study retrospectively evaluates all URTOR events within 30 days after neurosurgical procedures at our center, with two descriptive aims: (1) to characterize temporal patterns of URTOR by examining early (≤7 days) and late (8–30 days) events, and (2) to describe their etiological categories and distribution across surgeon experience. The early/late distinction is not intended as a new classification system but as a framework to illustrate how different mechanisms of reoperation tend to cluster within the postoperative period. By presenting these patterns, we aim to provide clinically relevant observations that may inform surgical training and quality improvement.

## 2. Materials and Methods

### 2.1. Inclusion and Exclusion Criteria

A total of 18,258 neurosurgical procedures performed between January 2010 and December 2018 at our hospital, one of the major academic referral hospitals in Izmir, were retrospectively analyzed from patient medical records. Patients who underwent URTOR within the first 7 days were referred to as early URTOR, and all patients who underwent URTOR within 8–30 days were classified as late URTOR. The 7-day and 30-day cutoffs were adopted based on prior neurosurgical literature, where this division is commonly used to distinguish acute, surgery-related complications from delayed postoperative events [13,14]. The 30-day interval also represents the standard perioperative period commonly used for surgical quality assessment [15]. Operative duration and recovery may differ across neurosurgical subspecialties; however, a uniform temporal framework was applied to allow consistent comparisons across procedures. This early/late division should be regarded as a descriptive analytical approach rather than a prescriptive classification, which serves only to illustrate how different mechanisms of URTOR tend to cluster within the first postoperative month.

All patients who underwent URTOR within 30 days of the index surgery were included in the analysis at our clinic. The inclusion was independent of patient age (pediatric or adult), surgical urgency (elective or emergency), or index surgery type. The exclusion criteria included the following: (1) index surgery of patients performed at another center, (2) patients who underwent unplanned re-operation after postoperative 30 days, (3) procedures performed with local anesthesia, and (4) procedures that are planned before or during the index operation, which are referred to as a planned return to the operating room. Junior surgeons were defined as those with ≤5 years of residency experience or ≤2 years of independent practice, whereas “senior” surgeons had experience exceeding these thresholds. Junior surgeons never entered any surgery without a senior observer beside them.

### 2.2. Index Surgical Procedures of Reoperated Patients

The patients’ index operations were categorized as cerebrospinal fluid (CSF) fistula repair, external ventricular drainage (EVD), meningomyelocele, cranial trauma operation, spinal instrumentation, kyphoplasty/vertebroplasty, ventriculo–peritoneal (V-P) shunt, endoscopic third ventriculostomy (ETV), intracranial tumor excision, intracerebral hematoma (ICH), aneurysm clipping, spinal simple decompression, decompressive craniectomy, chronic subdural hematoma, and cerebral abscess.

### 2.3. Classification of URTOR by Cause

URTOR Surgery Type denoted the specific indication for reoperation, defined according to the main finding or procedure performed (e.g., epidural hematoma, shunt revision, wound infection). To provide a clearer overview and a broader perspective on reoperation patterns, these surgeries were subsequently grouped into broader categories under the URTOR Classification. Each URTOR event was classified into one of the following five categories based on the primary reason for reoperation: (1) hemorrhagic and vascular complications, which included ICH, subdural hematoma, epidural hematoma, decompressive hemicraniectomy, spinal epidural hematoma, and rebleeding; (2) infectious complications, which included wound and surgical cavity infection; (3) CSF complications, which included shunt revision, CSF leak, ETV failure, acute hydrocephalus, and V-P shunt after EVD/meningomyelocele repair; (4) technical or procedure-related complications, which included revision of spinal instrumentation, wrong-level operation, residual tumor, aneurysm residue, insufficient spinal decompression, and foreign body; and (5) supportive procedures, which included tracheostomy. This categorization was designed to preserve the cohort’s procedural diversity while improving interpretability and allowing clearer visualization of overall trends and more meaningful statistical comparisons.

To minimize classification bias, all URTOR cases were independently reviewed by two neurosurgeons who were not involved in the index surgeries. Discrepancies in classification were resolved through consensus discussion with senior faculty members. Although formal interobserver reliability testing was not performed, this multi-reviewer approach was intended to enhance consistency and reduce subjective bias in assigning surgeon-attributable complications.

### 2.4. Patient and Surgical Variables Analyzed

The groups were then analyzed for sex, age, primary diagnosis, surgeon seniority, reason for URTOR, and the effect of operations performed on weekends on URTOR rates. Differences between the groups were investigated.

### 2.5. Statistical Analyses

SPSS 25.0 (IBM Corporation, Armonk, NY, USA) was used to analyze the variables. The conformity of the data to a normal distribution was evaluated using the Kolmogorov–Smirnov and Shapiro–Wilk tests. The Mann–Whitney U test with Monte Carlo results was used to compare two independent groups according to the quantitative variables. Pearson’s chi-square, Fisher’s exact, and Fisher–Freeman–Halton tests were performed using the Monte Carlo Simulation technique for the comparison of categorical variables, and the comparison of column ratios was expressed as Benjamini–Hochberg-corrected *p*-values. Quantitative variables were expressed as mean (standard deviation) and median (first to third quartile), whereas categorical variables were expressed as *n* (%). Variables were analyzed at a 95% confidence level, and *p*-values < 0.05 were considered significant.

## 3. Results

### 3.1. Patient Demographics and URTOR Characteristics

In total, 324 URTORs were identified among 18,258 procedures (1.8%). The mean age of patients was 34.7 ± 27.7 years, and the median age was 38 years (IQR: 1–58). Most cases (37.0%, *n* = 120) were children (<18 years), 47.2% (*n* = 153) were adults (18–65 years), and 15.7% (*n* = 51) were older (>65 years). Of the index operations, 89.5% (*n* = 290) were performed during working hours. The most common index operations were V-P shunt (19.1%, *n* = 62), cranial tumor surgery (16.7%, *n* = 54), and spinal instrumentation (16.0%, *n* = 52). Demographic and clinical characteristics of the patients and detailed data regarding the index surgery and URTOR are presented in Table 1.

### 3.2. Comparison of Early and Late URTOR Groups

A significant difference was observed in the median age between the late and early URTOR groups (*p* < 0.001); patients in the early group (median 46, IQR: 15–62) were significantly older than those in the late group (median 22, IQR: 0.33–52) (Table 2). Age group distribution also differed significantly between the groups (*p* = 0.001); the proportion of adult patients was significantly higher in the early URTOR group (54.5%), whereas the proportion of pediatric patients was significantly higher in the late URTOR group (49.6%). No significant difference was observed between the groups in terms of sex distribution (*p* = 0.648) or the day of the index surgery (weekday/weekend) (*p* = 0.854).

The distribution of the index surgery types showed a significant difference between the two groups (*p* = 0.004). In particular, the rate of late URTORs was significantly higher in patients who underwent V-P shunt (24.8% vs. 15.2%) and ETV (6.0% vs. 1.0%) as index surgery compared to those in the early URTOR group.

When the distribution of the URTORs was analyzed by classification, CSF-related (34.6%) and hemorrhagic/vascular (31.4%) complications were the most common in the early URTOR group. In contrast, CSF-related complications (51.1%) were the most common cause in the late URTOR group, followed by infectious (19.5%) and hemorrhagic/vascular (18.8%) complications. Significant differences were also observed in the detailed analysis according to classification. Hemorrhagic and vascular complications were significantly associated with pediatric patients (40.0% vs. 11.7%; *p* = 0.006), and the index surgery was chronic subdural hematoma (56.0% vs. 21.7%; *p* = 0.033) in late URTOR. Early URTOR was significantly associated with adult patients (65.0% vs. 36.0%) in this group.

Among the CSF-related complications, the median age of the late URTOR group (1 year) was significantly lower than that of the early URTOR group (9.5 years) (*p* = 0.023). No significant differences were observed between the early and late URTOR groups in terms of demographic characteristics or index surgery type in the infectious and surgery-related complication groups (*p* > 0.05) (Table 2).

### 3.3. Prevalence of Senior vs. Junior Surgeons in URTOR

In the late URTOR group, 74.4% (*n* = 99) of cases had their index operation performed by junior surgeons, compared to only 29.8% (*n* = 57) in the early URTOR group. In contrast, 70.2% (*n* = 134) of the patients in the early URTOR group had their index operation performed by senior surgeons, compared to 25.6% (*n* = 34) in the late URTOR group. Therefore, the likelihood of late URTOR after operations performed by junior surgeons was significantly higher than that of senior surgeons (*p* < 0.001; Table 2).

In the hemorrhagic and vascular complication group, the distribution of surgeon experience level differed significantly between early and late URTOR cases (*p* < 0.001), with a junior/senior ratio of 50.0%/50.0% in early URTOR compared to 56.0%/44.0% in late URTOR. Similarly, in the infectious complications group, the proportion of index surgery performed by a junior surgeon was statistically higher in late URTOR cases (69.2%) (*p* < 0.001). The same pattern was observed in the CSF-related complications group, where the proportion of late URTOR cases operated on by junior surgeons (76.5%) was significantly higher than early URTOR cases (25.8%) (*p* < 0.001). In contrast, no significant difference was observed between surgeon experience level and URTOR timing (early vs. late) in the surgery-related complication (*p* = 0.100) and supportive procedure groups.

Recurrent bleeding was a significantly more prevalent cause in junior surgeons’ early URTORs (38.6%) compared to late URTORs (17.2%). In contrast, the recurrent bleeding rate in senior surgeons’ cases was generally low (1.5% early, 0% late), and no significant difference was noted between early and late URTOR. Similarly, the decompressive hemicraniectomy rate (5.3%) in junior surgeons’ early URTOR cases was significantly higher than in late URTOR cases; these specific early URTOR causes did not stand out in senior surgeons’ cases. In contrast, ICH was significantly more common in early URTORs (11.9%) than in late URTORs (0%) among senior surgeons, whereas no significant difference was observed in ICH rates between the early and late stages among junior surgeons.

Acute hydrocephalus significantly increased in junior surgeons’ cases in late URTOR (11.1%) compared with early URTOR (0%). In both experience groups, wound site infection was a significantly more common cause of URTOR in the late period than in the early period (junior: 12.1% late vs. 1.8% early; senior: 17.6% late vs. 6.0% early). However, ETV insufficiency showed a significant increase in the late period (23.5%) compared with the early period (1.5%) only in the cases of senior surgeons (Table 3).

### 3.4. Relationship Between Patient Characteristics, Index Surgeries, and URTOR Classification

URTORs in the pediatric age group were significantly more frequently classified as CSF-related complications (59.7%) compared to other URTOR types (hemorrhagic/vascular, infectious, surgical-related, and supportive procedures) (Table 4). In adult patients, hemorrhagic and vascular complications (56.5%) and surgeon-related complications (67.9%) were significantly more common than CSF-related complications. In older patients, hemorrhagic and vascular complications (23.5%) were also significantly higher than CSF-related complications. Sex (*p* = 0.285) and the day of the week on which the index surgery was performed (weekday/weekend) (*p* = 0.628) had no significant effects on URTOR classification. The significant association between the index surgery type and the URTOR classification (*p* < 0.001) was detailed in the previous section (Table 4). The distribution of URTOR etiologies varied according to the type of index surgery (see Table S1 for the complete dataset).

## 4. Discussion

A total of 18,258 neurological surgeries were evaluated in this study, and URTOR developed in 324 patients. The rates vary in the literature and URTORs are closely associated with mortality, as they introduce additional risks to the surgical process [7,12,13,16]. Wang et al. reported a mortality rate of 8% in URTORs [1]. In a 2022 study on glioma surgeries, a 3.2% rate was reported in URTORs [17]. The lower mortality rate in the current study is likely due to the fact that other studies focused on cranial cases, whereas this study included both spinal and cranial cases.

Although the timing of URTOR differs in the literature, such as the first 7 or 30 days, URTORs that develop after the first week may have a different etiology [1]. Huang et al. were the first to classify URTORs as early or late. Additionally, hemorrhagic URTORs were predominant in the early stages, whereas CSF-related cases were predominant in later stages, similar to the results of this study [14]. Moreover, a significant majority of cases (59.0%) occurred within the first 7 days post-surgery, whereas 41.0% occurred between 8 and 30 days, consistent with the results of previous studies. However, in their series of 6912 neurosurgical procedures, McLaughlin et al. reported that shunt malfunction was the most frequent cause of early unplanned reoperation within the first 7 postoperative days [13]. Our study demonstrated a predominance of shunt-related URTORs in the late postoperative period. This discrepancy may be explained by differences in patient composition and etiology. While early shunt failures in McLaughlin’s cohort were largely due to intraoperative technical issues or immediate mechanical obstruction, the late shunt revisions in our series were mainly associated with delayed infection, progressive catheter obstruction, or peritoneal fibrosis, which typically manifest after the first week. Moreover, because our cohort included more pediatric patients, shunt malfunction might have appeared later, as children can tolerate increased intracranial pressure longer due to open cranial sutures. In their 30-day URTOR analysis, Wang et al. reported that 24 of 62 patients (38.7%) required reoperation within the first 24 h after surgery [1]. Across the entire 30-day period, postoperative rebleeding was the most frequent cause of URTOR, accounting for 45 of 70 cases (64.3%), including 55.7% recurrent and 8.6% new hematomas. The authors identified inadequate intraoperative hemostasis as the principal factor contributing to rebleeding.

V-P shunt dysfunction was the most common URTOR cause in the late group. Several factors cause shunt dysfunction. For example, IVH, previous EVD, and CSF alterations had a significant association with the development of shunt failure at 30 days postoperatively [18]. In the current study, 26 cases in the early group and 30 cases in the late group had shunt dysfunction-related URTOR. Of these, 23 of 56 (41.07%) had previously undergone EVD, 9 of 56 (16.07%) had IVH, and in 24 (42.85%), no facilitating etiologic cause was found.

When comparing the early and late URTOR groups, a significantly lower average and median age were observed in the late group, particularly among pediatric patients with hemorrhagic/vascular complications. Hemorrhagic/vascular complications were more common in the early group; however, pediatric cases occurred more often in the late group. Moreover, the median age for CSF-related complications was significantly lower in pediatric patients undergoing late URTOR than in those undergoing early URTOR. This finding may be attributed to the limited ability of children to adequately verbalize and communicate clinical symptoms compared with adults. Pediatric V-P shunt procedures are frequently performed at our center. Because the elasticity of children’s skulls and the persistence of patent sutures enable them to accommodate elevated intracranial pressure for an extended time, signs of increased intracranial pressure often emerge in a relatively subacute period [7]. Consistent with these findings, 40% of URTORs were observed between 8 and 30 days, and the median shunt failure was 9 days. Similarly, Donoho et al. highlighted the silent course of many shunt failures, showing that V-P-shunt revisions often arise beyond the early postoperative window rather than immediately after surgery [19]. Furthermore, junior surgeons performed most index operations that later required CSF-related URTOR—76.5% of late cases—whereas their share in early URTORs was only 25.8%. Given the technical complexity of V-P shunt placement, even small oversights can lead to late failure, especially among less experienced surgeons.

In our study, 74.4% of patients who developed URTOR in the late period had their initial surgical procedure performed by a surgeon with lower experience, whereas this rate was only 29.8% in URTOR cases that occurred in the early period; in contrast, 70.2% of early URTOR cases were performed by senior surgeons. This inversion suggests that delayed technical or postoperative issues, rather than acute catastrophes that typically drive early reinterventions, emerge more frequently when the primary surgeon is still mastering operative nuances.

In the hemorrhagic/vascular subgroup, the junior/senior distribution shifted from 50:50 in the early stage to 56:44 in the late stage, indicating that urgent bleeding cases partially offset the differences in experience. These findings do not provide causal evidence but rather highlight the level of experience as a potential risk factor for late URTOR, thereby emphasizing the need for close mentoring and rigorous postoperative follow-up for training surgeons. A multicenter analysis reported that surgical experience level (from 0–5 years to ≥21 years) did not significantly affect the risk of URTOR in five high-volume neurosurgical procedures [20]. In contrast, a study involving 4516 neurosurgical patients in China indicated that surgical experience significantly affects unplanned reoperation rates, with senior surgeons (>10 years of experience) having significantly lower reoperation rates than junior surgeons [1]. Late URTORs occur predominantly in cases performed by junior surgeons; however, this relationship may not be universal; therefore, supervision protocols must be considered when generalizing the findings.

Infection is unexpected in the early postoperative period; however, its prevalence among junior surgeons suggests several possible mechanisms. First, minor deviations from consistent application of aseptic and antiseptic rules increase the risk of surgical site infection (SSI), and this risk may be higher in less experienced surgeons [21,22]. Second, the likelihood of infection increases gradually with each additional hour of surgery [23]. Third, emergency procedures have significantly higher SSI rates than elective procedures. In our clinic, the fact that junior surgeons are more involved in emergency procedures than senior surgeons may contribute to this infection risk [24]. In addition, our intra-operative experience indicates that less experienced surgeons, often hurrying to secure hemostasis during the initial exposure, rely more heavily on monopolar electrocautery, resulting in unnecessary tissue necrosis and predisposing the wound to infection.

In this study, recurrent bleeding accounted for 38.6% of early URTORs in junior-led cases but only 17.2% of their late URTORs. Postoperative hemorrhage is the most common cause of URTOR, and surgeon experience may significantly reduce its frequency [1]. Furthermore, in a study focusing on intracerebral hemorrhage surgery, the surgeon’s experience was the strongest modifiable predictor of postoperative rebleeding. Senior surgeons achieved lower postoperative rebleeding rates by spending more time on meticulous hemostasis [25]. These findings indicate that the prevention of early hemorrhagic URTOR relies on precise hemostatic techniques and sound intraoperative judgment. However, the results of this study show that junior status is not an independent predictor of overall bleeding risk, which suggests that hemorrhage accounts for a relatively larger share of the causes when a junior-led case returns to the operating room.

Spinal instrumentation revision was the most prominent subgroup of technical or procedure-related complications. Nearly all cases that required reoperation in the early period of spinal revision surgery were revised because of pedicle screw malposition detected by routine computed tomography (CT); only one patient had a subfascial drain fracture removed. In patients who were revised after the seventh day, mild screw misalignments that were initially considered tolerable caused severe pain or neurological deficits during mobilization. Surgical site infection associated with spinal implants is the primary cause of URTORs, and a significant proportion of cases require sequential debridement and device replacement [26]. Similarly, a large-scale study examining implant-related mechanical complications demonstrated that screw loosening or malposition accounted for a significant portion of the revision load [27]. This study showed that all instrumentation revisions within the first 30 days occurred in cases led by senior surgeons, an unexpected finding given that our trainees begin spinal fixation early in their learning curve. Several factors could explain this pattern. First, when junior surgeons operate under senior supervision, attending senior surgeons act as an “extra set of eyes,” guiding screw orientation and intervening at the slightest concern about malposition, likely lowering the need for later revision. Second, junior surgeons tended to be more cautious and proactive when they were the primary operators, further reducing errors. Notably, our operating rooms currently rely on standard C-arm fluoroscopy. The future adoption of O-arm imaging, intra-operative CT, or spinal navigation systems could decrease screw-related revision rates by providing real-time, three-dimensional confirmation of instrument placement.

One wrong-level surgery was a case of vertebroplasty applied to a thoracic vertebral fracture. The reason for the wrong-level surgery was that morbidly obese patients had multiple-level fractures. The operation team could not evaluate the chronic compressions in the lower and upper neighborhoods of the fracture under the preoperative scope, which was clearly observed on magnetic resonance imaging. The other three cases had high-level lumbar discopathies, and all three patients had advanced degenerative scoliosis. The surgical team stated that when they counted separately in the coronal and sagittal planes, they thought they had reached different vertebrae. Morbidly obese patients and those with scoliosis-related abnormal vertebrae may experience wrong-level surgeries [28]. A systematic review of wrong-level surgery that included two studies on pure lumbar spine surgeries indicated that the wrong-level surgery incidence was 0.1–15% [29]. This rate for our center was 0.24%, and one of the patients included in this study was morbidly obese. Ammerman et al. reported that being over 55 years old and having a pathology above L5-S1 are associated with wrong-level surgery [30]. Epstein et al. also highlighted that morbid obesity increases perioperative morbidity/mortality, including wrong-level surgery [31].

Three patients underwent URTOR because of foreign bodies in the surgical area. Two cases involved spinal surgeries, and one involved an endoscopic transsphenoidal approach. The patient who underwent endoscopic transsphenoidal surgery for a sellar tumor was reexplored because the discarded cotton pad was forgotten at the operation site. During the endoscopic approach, the aspirator that entered the nose was aspirated, and the rope of the cotton pad was cut. Therefore, the cotton watered with blood is lost after the procedure. Especially in endoscopic transsphenoidal approaches, the aspirator may aspirate the ropes of cotton pads during repetitive entries into the nostrils, which may cut the rope. As the surgeon continuously looks at the monitor during the operation, the escape of the rope into the aspirator can be overlooked during the aspirator entrance. Therefore, more attention should be paid to this issue.

### 4.1. Reoperate or Manage Conservatively?

Choosing between reoperation and conservative management is a key part of postoperative decision-making. In patients with postoperative CSF leakage, we evaluate the options of acetazolamide and lumbar drainage before considering surgical re-exploration. Our clinical experience suggests that most cases can be treated with these approaches. Barber et al. reported that lumbar drainage used for 3–5 days, with daily drainage volumes of 120–360 mL, achieved success rates of 90–92% for spinal CSF fistulas [32]. Preoperative lumbar drainage may also be considered in cases where a postoperative leak is anticipated.

In patients with acute hydrocephalus or those with intracranial hemorrhage accompanied by a midline shift, conservative management was not considered due to the life-threatening condition. However, in cases of mass lesions causing obstructive hydrocephalus, preoperative placement of an EVD instead of an immediate VP shunt often prevented postoperative shunt dependency.

Postoperative intracerebral hematomas were managed according to commonly accepted criteria. Surgery was indicated for hematomas larger than 3 cm, those associated with neurological deterioration, brainstem compression, or hydrocephalus due to ventricular obstruction. Patients for whom surgery was not indicated were managed with anti-edema therapy, antiepileptic medication, and supplementation with vitamins C and E, along with close neurological and radiological follow-up. In the literature, hematomas presenting with neurological deficits are generally treated with immediate reoperation, whereas small and asymptomatic ones are managed with close observation [33]. Our practice followed the same principle.

For residual aneurysms, the 2023 AHA/ASA guidelines emphasize complete obliteration as the primary goal. When this cannot be safely achieved in the acute phase, securing the rupture site partially and considering delayed retreatment—either microsurgical or endovascular—is considered reasonable [34]. Yu et al. compared clipping, coiling, and conservative follow-up in patients with residual or recurrent aneurysms and reported that good functional outcomes can also be achieved with observation in selected cases, although retreatment rates differed among the treatment groups [35]. In our practice, if the residual portion appears calcified or stable and measures less than 2 mm, we recommend close radiological surveillance and thorough patient counseling rather than immediate retreatment. However, the final treatment decision is made through a multidisciplinary discussion with the interventional neuroradiology team.

Tumor management in our series depended on the expected pathology and intraoperative frozen findings. In patients with a presumed diagnosis of glioblastoma, maximal safe resection was prioritized in younger and pediatric cases, whereas in elderly or frail patients, the main goal was to minimize surgical risk and achieve adequate decompression to relieve pressure on vital neurovascular structures. Previous studies have reported that the decision for early repeat resection in glioblastoma differs across centers, with the extent of resection threshold of ≥70–98% being a key prognostic factor [36]. Damante et al. demonstrated that performing early repeat resection within eight weeks and achieving ≥95% extent of resection resulted in oncologic outcomes comparable to those of patients who initially reached near-total resection, without compromising the timing of adjuvant therapy [37].

In cases of VP shunt infection, when cultures obtained from the shunt reservoir indicated infection, the entire system was removed, an EVD was placed, and targeted antibiotic therapy was initiated. A systematic review by Robinson et al. supported this approach, showing that shunt removal combined with EVD placement and antibiotic treatment is associated with higher success rates, whereas antibiotic therapy alone carries a greater risk of failure and recurrence [38].

Although surgical debridement was performed aggressively in cases of wound infection, patients were evaluated daily in consultation with the infectious disease team, and decisions regarding reoperation were made through a multidisciplinary approach.

In spinal screw malposition cases, management was based on symptoms, breach size, and direction. Medial breaches smaller than 2 mm were considered safe, while those exceeding this threshold—particularly ≥4 mm—were associated with a higher risk of neurological injury and often required revision [39]. Lateral or anterior breaches were generally left untreated if asymptomatic, but early correction was preferred when there was foraminal or anterior vascular proximity, especially in the thoracic region where major vessel or visceral injury could occur [40].

Symptomatic patients with new radiculopathy, weakness, or significant pain underwent early revision. According to Dunn et al., asymptomatic breaches can be safely managed with clinical or CT follow-up, and routine CT is not necessary in all asymptomatic cases [41]. However, in our practice, postoperative CT was routinely performed after spinal instrumentation to confirm screw placement.

### 4.2. Influence of Comorbidities, Case Complexity, and Surgical Urgency

Our retrospective database lacked standardized comorbidity fields; nevertheless, prior evidence indicates that increased comorbidity burden is associated with higher postoperative complication and URTOR rates [42]. In our series, we similarly observed that many reoperated chronic subdural hematoma patients who developed rebleeding were those requiring antiaggregant therapy, and that degenerative scoliosis in spinal instrumentation cases often posed orientation challenges for junior surgeons. These observations underline the importance of performing such operations collaboratively with experienced team members. Furthermore, in emergency procedures, some routine safety steps may occasionally be omitted, which potentially increases the risk of infection or reoperation [7].

Although intraoperative fluoroscopy is routinely available in our operating rooms, both ventricular shunt placement and spinal screw fixation are often performed using freehand techniques. Consequently, some cases with complex or distorted anatomy may inherently carry a higher risk of reoperation due to suboptimal trajectory or implant positioning. The wider adoption of intraoperative neuronavigation in such patients could therefore help reduce preventable reoperations [43]. Additionally, as intraoperative indocyanine green angiography is not yet available in our center, small residual aneurysms may occasionally go undetected despite careful microscopic inspection. Performing these procedures under indocyanine green angiography guidance, combined with detailed preoperative three-dimensional vascular reconstructions, may enhance anatomical accuracy and further decrease the likelihood of unplanned reoperations [44,45].

Institutional factors may also underlie the experience-related patterns observed. At our center, junior surgeons operate under senior supervision, yet the degree of oversight can fluctuate with case load, emergency demand, or time of day. These variations may help explain the greater proportion of late URTORs following procedures led by less experienced surgeons. Likewise, postoperative follow-up and infection-control routines, though standardized, can be affected by staffing and workload during high-volume or night shifts. Strengthening supervision models and maintaining consistent postoperative care standards could further reduce preventable reoperations.

The current literature on URTOR is fragmented. Many studies focus only on specific areas, such as pediatric cohorts, spinal surgery, or short observation periods [7,8,9]. While such specific studies provide detailed results within their own contexts, a broader perspective can be obtained in a series that evaluates different disciplines together. Previous studies have examined the impact of surgeon experience [1,6], whereas others have specifically assessed early unplanned reoperations and their preventability as a potential quality indicator [13]. However, none of these have focused cranial, spinal, pediatric, and adult cases simultaneously. Additionally, previous reports have generally presented causes as scattered lists of complications without using a general classification framework.

To our knowledge, this is the first study to differentiate early from late URTORs, examine outcomes in relation to surgeon experience, and apply a structured five-category etiological classification, thereby offering a comprehensive and consistent framework for understanding URTOR across a broad spectrum of neurosurgical procedures.

### 4.3. Limitations

This study has some limitations. First, its single-center and retrospective design limits the generalizability of the findings, as they may only reflect our hospital’s patient profile and the surgical interventions performed at our institution. Another limitation is that comorbidities and patient-related risk factors were not within the scope of this analysis. Nonetheless, these variables are known to influence postoperative outcomes and should be incorporated into future studies to better delineate their role in URTOR. In addition, Exact *p*-values smaller than 0.001 were reported conventionally as “*p* < 0.001,” since the statistical software output indicated 0.000; this follows standard reporting practice in biomedical research. Logistic regression analysis was attempted to control for potential confounders such as age and case type; however, no valid models could be obtained due to data distribution constraints.

The time-based classification used in this study (≤7 days vs. 8–30 days) is another limitation. Although these intervals are consistent with patterns described in previous neurosurgical reoperation studies, they are not universally standardized and should be interpreted as descriptive rather than definitive. Postoperative trajectories may vary substantially across neurosurgical subspecialties, and maybe a single time-based framework cannot fully account for this heterogeneity. Accordingly, dividing events into ‘early’ and ‘late’ may oversimplify the postoperative continuum and introduce a degree of interpretive uncertainty.

## 5. Conclusions

This study showed that hemorrhagic–mechanical complications are prominent in early-stage URTORs. In contrast, infection and CSF-related problems are prominent in the late stage. Additionally, in the later period, most cases leading to URTOR involve junior surgeons. These results provide novel insights into targeted education and preventive strategies within the institution by identifying patient groups with a relatively higher risk of reoperation within each timeframe. To reduce URTOR rates, identifying the source of the problem in a precise surgical department, such as the neurosurgery department, will be possible with appropriate planning and special attention. Ensuring patient discharge after the procedure will also likely increase satisfaction. Beyond these observations, our findings also have practical implications. Incorporating URTOR analyses into residency training, supervision, and quality monitoring programs could help address preventable causes and strengthen clinical safety. Moreover, integrating URTOR data into quality-improvement dashboards would enhance continuous feedback and reinforce translational value. However, broader multicenter series and prospective studies are required to support these results and to develop general recommendations.

## Figures and Tables

**Table 1:** Demographic features, index procedures, re-operation indications, complication classes and timing for the 324 patients who underwent an unplanned return to the operating room (URTOR). Continuous variables are presented as mean ± SD and median (Q1–Q3); categorical variables as number (%).

	Mean (SD)	Median (Q1–Q3)
**Age**	34.7 (27.7)	38 (1–58)
	***n***	**%**
**Age Group**		
Child	120	37.0%
Adult	153	47.2%
Older Adult	51	15.7%
**Sex**		
Male	186	57.4%
Female	138	42.6%
**Day of Surgery**		
Weekday	290	89.5%
Weekend	34	10.5%
**Index Surgery Type**		
V-P shunt	62	19.1%
Cranial Tumor	54	16.7%
Spinal instrumentation	52	16.0%
Chronic subdural hematoma	31	9.6%
Cranial trauma operation	24	7.4%
Aneurysm clipping	24	7.4%
ICH	15	4.6%
Spinal simple decompression	13	4.0%
ETV	10	3.1%
EVD	10	3.1%
CSF fistula repair	7	2.2%
Meningomyelocele	7	2.2%
Decompressive craniectomy	7	2.2%
Cerebral abscess	6	1.9%
Kyphoplasty/vertebroplasty	2	0.6%
**URTOR Surgery Type**		
Shunt revision	56	17.3%
Acute Hydrocephalus	47	14.5%
Rebleeding	41	12.7%
Revision of Spinal Instrumentation	36	11.1%
Wound Infection	27	8.3%
ICH	25	7.7%
CSF leak	19	5.9%
Surgical Cavity Infection	12	3.7%
ETV Failure	10	3.1%
Tracheostomy	10	3.1%
Residual Operation	8	2.5%
EDH	8	2.5%
Decompressive Hemicraniectomy	5	1.5%
Insufficient Spinal Decompression	5	1.5%
Wrong Level Operation	4	1.2%
SDH	4	1.2%
Spinal EDH	2	0.6%
Foreign Body	2	0.6%
VP-Shunt after EVD/MMC	2	0.6%
Aneurysm residue	1	0.3%
**URTOR Classification**		
Cerebrospinal Fluid–Related Complications	134	41.4%
Hemorrhagic and Vascular Complications	85	26.2%
Technical or procedure-related complications	56	17.3%
Infectious Complications	39	12.0%
Supportive Procedures	10	3.1%
**Surgeon Experience Level**		
Junior	156	48.1%
Senior	168	51.9%
**URTOR Timing**		
Early	191	59.0%
Late	133	41.0%
**Index Surgery Type**	**Most frequent URTOR indication**	
V-P shunt	Shunt revision	
Cranial Tumor	CSF Diversion	
Spinal instrumentation	Revision of Spinal Instrumentation	
Chronic subdural hematoma	Rebleeding	
Cranial trauma operation	Rebleeding	
Aneurysm clipping	CSF Diversion	
ICH	Rebleeding	
Spinal simple decompression	Wound Infection	
ETV	ETV Failure	
EVD	ICH	
CSF fistula repair	CSF Leak	
Meningomyelocele	Wound Infection	
Decompressive craniectomy	CSF Leak	
Cerebral abscess	Surgical Cavity Infection	
Kyphoplasty/vertebroplasty	Wrong Level Operation	
	**Mean (SD)**	**Median (Q1–Q3)**
**Interval Between Index Surgery and URTOR (Days)**	7.9 (7.1)	6 (2–12)

SD, standard deviation; V-P, ventriculoperitoneal; ETV, endoscopic third ventriculostomy; EVD, external ventricular drainage; ICH, intracerebral hemorrhage; EDH, epidural hematoma; SDH, subdural hematoma: CSF cerebrospinal fluid. *Junior surgeons* = residents with ≤5 years of training or ≤2 years independent practice; *Senior surgeons* = experience beyond this threshold. *Early URTOR* = ≤7 days after the index procedure; *Late URTOR* = 8–30 days.

**Table 2:** Comparison of early and late URTOR groups by complication classes and clinical variables.

	URTOR Classification															Total (*n* = 324)		*p*
	Hemorrhagic and Vascular Complications		*p*	Infectious Complications		*p*	Cerebrospinal Fluid–Related Complications		*p*	Technical or Procedure-Related Complications		*p*	Supportive Procedures		*p*			
	URTOR Timing			URTOR Timing			URTOR Timing			URTOR Timing			URTOR Timing			URTOR Timing		
	Early (*n* = 60)	Late (*n* = 25)		Early (*n* = 13)	Late (*n* = 26)		Early (*n* = 66)	Late (*n* = 68)		Early (*n* = 42)	Late (*n* = 14)		Early (*n* = 10)	Late (*n* = 0)		Early (*n* = 191)	Late (*n* = 133)	
**Age, median (Q1–Q3)**	54 (41–64.5)	27 (1–65)	**0.073 ^u^**	44 (40–61)	28.5 (1–56)	0.239 ^u^	9.5 (0.5–54)	1 (0.25–35)	**0.023 ^u^**	52.5 (33–63)	47.5 (29–61)	0.487 ^u^	41.5 (20–60)	-	-	46 (15–62)	22 (0.33–52)	**<0.001 ^u^**
**Age Group, *n* (%)**			**0.006 ^c^**			0.074 ^ff^			0.548 ^c^			0.800 ^ff^			-			**0.001 ^c^**
Child	7 (11.7)	**10 (40.0) A**		1 (7.7)	10 (38.5)		37 (56.1)	43 (63.2)		7 (16.7)	3 (21.4)		2 (20.0)	-		54 (28.3)	**66 (49.6) A**	
Adult	**39 (65.0) B**	9 (36.0)		10 (76.9)	10 (38.5)		21 (31.8)	20 (29.4)		28 (66.7)	10 (71.4)		6 (60.0)	-		**104 (54.5) B**	49 (36.8)	
Older Adult	14 (23.3)	6 (24.0)		2 (15.4)	6 (23.1)		8 (12.1)	5 (7.4)		7 (16.7)	1 (7.1)		2 (20.0)	-		33 (17.3)	18 (13.5)	
**Sex, *n* (%)**			0.484 ^c^			0.734 ^c^			0.600 ^c^			0.999 ^c^			-			0.648 ^c^
Male	34 (56.7)	12 (48.0)		8 (61.5)	13 (50.0)		36 (54.5)	41 (60.3)		25 (59.5)	8 (57.1)		9 (90.0)	-		112 (58.6)	74 (55.6)	
Female	26 (43.3)	13 (52.0)		5 (38.5)	13 (50.0)		30 (45.5)	27 (39.7)		17 (40.5)	6 (42.9)		1 (10.0)	-		79 (41.4)	59 (44.4)	
**Day of Surgery, *n* (%)**			0.074 ^c^			0.999 ^f^			0.239 ^f^			0.999 ^f^			-			0.854 ^c^
Weekday	55 (91.7)	19 (76.0)		12 (92.3)	24 (92.3)		58 (87.9)	64 (94.1)		37 (88.1)	13 (92.9)		8 (80.0)	-		170 (89.0)	120 (90.2)	
Weekend	5 (8.3)	6 (24.0)		1 (7.7)	2 (7.7)		8 (12.1)	4 (5.9)		5 (11.9)	1 (7.1)		2 (20.0)	-		21 (11.0)	13 (9.8)	
**Index Surgery Type, *n* (%)**			**0.033 ^ff^**			0.132 ^ff^			0.386 ^ff^			0.341 ^ff^			-			**0.004 ^ff^**
CSF fistula repair	-	-		-	-		5 (7.6)	2 (2.9)		-	-		-	-		5 (2.6)	2 (1.5)	
EVD	3 (5.0)	3 (12.0)		0 (0.0)	1 (3.8)		0 (0.0)	3 (4.4)		-	-		-	-		3 (1.6)	7 (5.3)	
Meningomyelocele	-	-		0 (0.0)	4 (15.4)		2 (3.0)	1 (1.5)		-	-		-	-		2 (1.0)	5 (3.8)	
Cranial trauma operation	9 (15.0)	2 (8.0)		0 (0.0)	1 (3.8)		1 (1.5)	2 (2.9)		1 (2.4)	1 (7.1)		7 (70.0)	-		18 (9.4)	6 (4.5)	
Spinal instrumentation	1 (1.7)	0 (0.0)		2 (15.4)	9 (34.6)		0 (0.0)	1 (1.5)		30 (71.4)	9 (64.3)		-	-		33 (17.3)	19 (14.3)	
Kyphoplasty/vertebroplasty	-	-		-	-		-	-		2 (4.8)	0 (0.0)		-	-		2 (1.0)	0 (0.0)	
V-P shunt	-	-		1 (7.7)	3 (11.5)		28 (42.4)	30 (44.1)		-	-		-	-		29 (15.2)	**33 (24.8) A**	
ETV	-	-		-	-		2 (3.0)	8 (11.8)		-	-		-	-		2 (1.0)	**8 (6.0) A**	
Cranial Tumor	16 (26.7)	3 (12.0)		3 (23.1)	0 (0.0)		14 (21.2)	10 (14.7)		4 (9.5)	4 (28.6)		-	-		37 (19.4)	17 (12.8)	
ICH	9 (15.0)	2 (8.0)		-	-		3 (4.5)	1 (1.5)		-	-		-	-		12 (6.3)	3 (2.3)	
Aneurysm clipping	6 (10.0)	0 (0.0)		-	-		8 (12.1)	6 (8.8)		1 (2.4)	0 (0.0)		3 (30.0)	-		18 (9.4)	6 (4.5)	
Spinal simple decompression	1 (1.7)	0 (0.0)		3 (23.1)	3 (11.5)		1 (1.5)	1 (1.5)		4 (9.5)	0 (0.0)		-	-		9 (4.7)	4 (3.0)	
Decompressive craniectomy	2 (3.3)	0 (0.0)		0 (0.0)	1 (3.8)		2 (3.0)	2 (2.9)		-	-		-	-		4 (2.1)	3 (2.3)	
Chronic subdural hematoma	13 (21.7)	**14 (56.0) A**		1 (7.7)	2 (7.7)		0 (0.0)	1 (1.5)		-	-		-	-		14 (7.3)	17 (12.8)	
Cerebral abscess	0 (0.0)	1 (4.0)		3 (23.1)	2 (7.7)		-	-		-	-		-	-		3 (1.6)	3 (2.3)	
**Surgeon Experience Level, *n* (%)**			**<0.001 ^f^**			**<0.001 ^f^**			**<0.001 ^c^**			**0.100 ^f^**			-			**<0.001 ^c^**
Junior	30 (50.0)	**24 (96.0)**		1 (7.7)	**18 (69.2)**		17 (25.8)	**52 (76.5)**		5 (11.9)	**5 (35.7)**		4 (40.0)	-		57 (29.8)	**99 (74.4)**	
Senior	**30 (50.0)**	1 (4.0)		**12 (92.3)**	8 (30.8)		**49 (74.2)**	16 (23.5)		**37 (88.1)**	9 (64.3)		6 (60.0)	-		**134 (70.2)**	34 (25.6)	

^u^ Mann–Whitney U Test (Monte Carlo), ^c^ Pearson Chi-Square Test(Monte Carlo), ^f^ Fisher Exact Test (Monte Carlo), ^ff^ Fisher Freeman Halton Test (Monte Carlo); Post Hoc Test: Benjamini–Hochberg correction, A: Significant according to Early UTOR group, B: Significant according to Late URTOR group, Q1: First Quartile, Q3: Third Quartile; **Hemorrhagic/vascular complications:** postoperative bleeding, intracerebral, subdural, or epidural hematoma, rebleeding, spinal epidural hematoma, and decompressive hemicraniectomy; **infectious complications:** wound infection and surgical cavity infection; **CSF-related complications:** shunt revision, CSF leak, ETV failure, acute hydrocephalus, and ventriculoperitoneal shunt after EVD or meningomyelocele repair; **Technical or procedure-related complications:** revision of spinal instrumentation, wrong-level surgery, residual tumor, aneurysm residue, insufficient spinal decompression, and retained foreign body; **supportive procedures**: tracheostomy BOS, cerebrospinal fluid; ETV, endoscopic third ventriculostomy; EVD, external ventricular drainage; ICH, intracerebral hemorrhage; EDH, epidural hematoma; SDH, subdural hematoma; Q1/Q3, first/third quartile. Surgeon experience definitions: *Junior* = ≤5 years of residency or ≤2 years independent practice; *Senior* = more experienced than these thresholds.

**Table 3:** Comparison of early and late URTOR groups according to surgeon experience, reoperation type, and complication classes. Data are presented as number (%). Statistical significance tested by Fisher–Freeman–Halton test with Monte Carlo simulation; significant pairwise differences indicated by Benjamini–Hochberg correction (A = significantly higher vs. early URTOR group, B = significantly higher vs. late URTOR group). *Junior* = ≤5 years residency or ≤2 years independent practice; *Senior* = beyond these thresholds.

	Total (*n* = 324)			Junior (*n* = 156)			Senior (*n* = 168)		
	URTOR Timing		*p*	URTOR Timing		*p*	URTOR Timing		*p*
	Early (*n* = 191)	Late (*n* = 133)		Early (*n* = 57)	Late (*n* = 99)		Early (*n* = 134)	Late (*n* = 34)	
	*n* (%)	*n* (%)		*n* (%)	*n* (%)		*n* (%)	*n* (%)	
**URTOR Surgery Type**			**<0.001 ^ff^**			**<0.001 ^ff^**			**<0.001 ^ff^**
Shunt revision	26 (13.6)	**30 (22.6) A**		15 (26.3)	30 (30.3)		11 (8.2)	0 (0.0)	
CSF leak	8 (4.2)	11 (8.3)		2 (3.5)	9 (9.1)		6 (4.5)	2 (5.9)	
Wound Infection	9 (4.7)	**18 (13.5) A**		1 (1.8)	**12 (12.1) A**		8 (6.0)	**6 (17.6) A**	
ETV Failure	2 (1.0)	**8 (6.0) A**		0 (0.0)	0 (0.0)		2 (1.5)	**8 (23.5) A**	
Revision of Spinal Instrumentation	**27 (14.1) B**	9 (6.8)		0 (0.0)	0 (0.0)		27 (20.1)	9 (26.5)	
Acute Hydrocephalus	30 (15.7)	17 (12.8)		0 (0.0)	**11 (11.1) A**		30 (22.4)	6 (17.6)	
Wrong Level Operation	4 (2.1)	0 (0.0)		2 (3.5)	0 (0.0)		2 (1.5)	0 (0.0)	
Residual Operation	3 (1.6)	5 (3.8)		0 (0.0)	5 (5.1)		3 (2.2)	0 (0.0)	
ICH	**20 (10.5) B**	5 (3.8)		4 (7.0)	5 (5.1)		**16 (11.9) B**	0 (0.0)	
SDH	2 (1.0)	2 (1.5)		0 (0.0)	2 (2.0)		2 (1.5)	0 (0.0)	
EDH	7 (3.7)	1 (0.8)		0 (0.0)	0 (0.0)		7 (5.2)	1 (2.9)	
Decompressive Hemicraniectomy	5 (2.6)	0 (0.0)		**3 (5.3) B**	0 (0.0)		2 (1.5)	0 (0.0)	
Aneurysm Reclipping	1 (0.5)	0 (0.0)		0 (0.0)	0 (0.0)		1 (0.7)	0 (0.0)	
Surgical Cavity Infection	4 (2.1)	8 (6.0)		0 (0.0)	6 (6.1)		4 (3.0)	2 (5.9)	
Insufficient Spinal Decompression	5 (2.6)	0 (0.0)		1 (1.8)	0 (0.0)		4 (3.0)	0 (0.0)	
Spinal EDH	2 (1.0)	0 (0.0)		1 (1.8)	0 (0.0)		1 (0.7)	0 (0.0)	
Surgical Foreign Body	2 (1.0)	0 (0.0)		2 (3.5)	0 (0.0)		0 (0.0)	0 (0.0)	
VP-Shunt after EVD/MMC	0 (0.0)	2 (1.5)		0 (0.0)	2 (2.0)		0 (0.0)	0 (0.0)	
Tracheostomy	**10 (5.2) B**	0 (0.0)		**4 (7.0) B**	0 (0.0)		6 (4.5)	0 (0.0)	
Rebleeding	24 (12.6)	17 (12.8)		**22 (38.6) B**	17 (17.2)		2 (1.5)	0 (0.0)	
**URTOR Classification**			**<0.001 ^ff^**			**<0.001 ^ff^**			0.009 ^ff^
Hemorrhagic and Vascular Complications	**60 (31.4) B**	25 (18.8)		**30 (52.6) B**	24 (24.2)		**30 (22.4) B**	1 (2.9)	
Infectious Complications	13 (6.8)	**26 (19.5) A**		1 (1.8)	**18 (18.2) A**		12 (9.0)	**8 (23.5) A**	
Cerebrospinal Fluid–Related Complications	66 (34.6)	**68 (51.1) A**		17 (29.8)	**52 (52.5) A**		49 (36.6)	16 (47.1)	
Technical or procedure-related complications	**42 (22.0) B**	14 (10.5)		5 (8.8)	5 (5.1)		37 (27.6)	9 (26.5)	
Supportive Procedures	**10 (5.2) B**	0 (0.0)		**4 (7.0) B**	0 (0.0)		6 (4.5)	0 (0.0)	

^ff^ Fisher Freeman Halton Test (Monte Carlo); Post Hoc Test: Benjamini–Hochberg correction, A: Significant according to Early UTOR group, B: Significant according to Late URTOR group; **Hemorrhagic/vascular complications:** postoperative bleeding, intracerebral, subdural, or epidural hematoma, rebleeding, spinal epidural hematoma, and decompressive hemicraniectomy; **infectious complications:** wound infection and surgical cavity infection; **CSF-related complications:** shunt revision, CSF leak, ETV failure, acute hydrocephalus, and ventriculoperitoneal shunt after EVD or meningomyelocele repair; **Technical or procedure-related complications:** revision of spinal instrumentation, wrong-level surgery, residual tumor, aneurysm residue, insufficient spinal decompression, and retained foreign body; **supportive procedures**: tracheostomy; URTOR, unplanned return to operating room; CSF, cerebrospinal fluid; ETV, endoscopic third ventriculostomy; EVD, external ventricular drainage; ICH, intracerebral hemorrhage; EDH, epidural hematoma; SDH, subdural hematoma; MMC, meningomyelocele.

**Table 4:** Comparison of patient demographics and index surgery characteristics across URTOR complication classes. Data presented as number (%). Statistical significance assessed by Fisher–Freeman–Halton test with Monte Carlo simulation. Pairwise significant differences indicated by Benjamini–Hochberg correction (*A* significantly higher vs. Hemorrhagic/Vascular; *B* Infectious; *C* CSF-related; *D* Technical or procedure-related complications; *E* Supportive Procedures).

	URTOR Classification					*p*
	Hemorrhagic and Vascular Complications	Infectious Complications	Cerebrospinal Fluid–Related Complications	Technical or Procedure-Related Complications	Supportive Procedures	
	*n* (%)	*n* (%)	*n* (%)	*n* (%)	*n* (%)	
**Age Group**						**<0.001 ^ff^**
Child	17 (20.0)	11 (28.2)	**80 (59.7) ABDE**	10 (17.9)	2 (20.0)	
Adult	**48 (56.5) C**	20 (51.3)	41 (30.6)	**38 (67.9) C**	6 (60.0)	
Older Adult	**20 (23.5) C**	8 (20.5)	13 (9.7)	8 (14.3)	2 (20.0)	
**Sex**						0.285 ^ff^
Male	46 (54.1)	21 (53.8)	77 (57.5)	33 (58.9)	9 (90.0)	
Female	39 (45.9)	18 (46.2)	57 (42.5)	23 (41.1)	1 (10.0)	
**Day of Surgery**						0.628 ^ff^
Weekday	74 (87.1)	36 (92.3)	122 (91.0)	50 (89.3)	8 (80.0)	
Weekend	11 (12.9)	3 (7.7)	12 (9.0)	6 (10.7)	2 (20.0)	
**Index Surgery Type**						**<0.001 ^ff^**
CSF fistula repair	0 (0.0)	0 (0.0)	7 (5.2)	0 (0.0)	0 (0.0)	
EVD	6 (7.1)	1 (2.6)	3 (2.2)	0 (0.0)	0 (0.0)	
Meningomyelocele	0 (0.0)	**4 (10.3) A**	3 (2.2)	0 (0.0)	0 (0.0)	
Cranial trauma operation	**11 (12.9) C**	1 (2.6)	3 (2.2)	2 (3.6)	**7 (70.0) AC**	
Spinal instrumentation	1 (1.2)	**11 (28.2) AC**	1 (0.7)	**39 (69.6) ABCE**	0 (0.0)	
Kyphoplasty/vertebroplasty	0 (0.0)	0 (0.0)	0 (0.0)	2 (3.6)	0 (0.0)	
V-P shunt	0 (0.0)	**4 (10.3) A**	**58 (43.3) ABD**	0 (0.0)	0 (0.0)	
ETV	0 (0.0)	0 (0.0)	10 (7.5)	0 (0.0)	0 (0.0)	
Cranial Tumor	19 (22.4)	3 (7.7)	24 (17.9)	8 (14.3)	0 (0.0)	
ICH	**11 (12.9) C**	0 (0.0)	4 (3.0)	0 (0.0)	0 (0.0)	
Aneurysm clipping	6 (7.1)	0 (0.0)	14 (10.4)	1 (1.8)	**3 (30.0) BD**	
Spinal simple decompression	1 (1.2)	**6 (15.4) AC**	2 (1.5)	4 (7.1)	0 (0.0)	
Decompressive craniectomy	2 (2.4)	1 (2.6)	4 (3.0)	0 (0.0)	0 (0.0)	
Chronic subdural hematoma	**27 (31.8) BCD**	3 (7.7)	1 (0.7)	0 (0.0)	0 (0.0)	
Cerebral abscess	1 (1.2)	**5 (12.8) C**	0 (0.0)	0 (0.0)	0 (0.0)	

^ff^ Fisher Freeman Halton Test (Monte Carlo); Post Hoc Test: Benjamini–Hochberg correction, A: Significant according to Hemorrhagic and Vascular Complications group, B: Significant according to Infectious Complications group, C: Significant according to Cerebrospinal Fluid–Related Complications group, D: Significant according to Technical or Procedure-related Complications group, E: Significant according to Supportive Procedures group; **Hemorrhagic/vascular complications:** postoperative bleeding, intracerebral, subdural, or epidural hematoma, rebleeding, spinal epidural hematoma, and decompressive hemicraniectomy; **infectious complications:** wound infection and surgical cavity infection; **CSF-related complications:** shunt revision, CSF leak, ETV failure, acute hydrocephalus, and ventriculoperitoneal shunt after EVD or meningomyelocele repair; **Technical or procedure-related complications:** revision of spinal instrumentation, wrong-level surgery, residual tumor, aneurysm residue, insufficient spinal decompression, and retained foreign body; **supportive procedures**: tracheostomy; URTOR, unplanned return to operating room; CSF, cerebrospinal fluid; ETV, endoscopic third ventriculostomy; EVD, external ventricular drainage; ICH, intracerebral hemorrhage; EDH, epidural hematoma; SDH, subdural hematoma; V-P, ventriculoperitoneal.

## Data Availability

The data presented in this study are not publicly available due to patient privacy and institutional policy restrictions. De-identified data may be made available from the corresponding author upon reasonable request and with permission of the institutional review board.

## Supplementary Materials

The following supporting information can be downloaded at: https://www.mdpi.com/article/10.3390/medicina61122117/s1, Table S1. Detailed dataset of patients who underwent URTOR.

## Abbreviations

The following abbreviations are used in this manuscript:

CSF	Cerebrospinal Fluid
CT	Computed Tomography
ETV	Endoscopic Third Ventriculostomy
EVD	External Ventricular Drainage
ICH	Intracerebral Hematoma
IVH	Intraventricular Hematoma
MRI	Magnetic Resonance Imaging
SPSS	Statistical Package for the Social Sciences
SSI	Surgical Site Infection
URTOR	Unplanned return to the operating room
VP Shunt	Ventriculoperitoneal Shunt
